# Analytical Approximant to a Quadratically Damped Duffing Oscillator

**DOI:** 10.1155/2022/3131253

**Published:** 2022-02-07

**Authors:** Alvaro H. Salas S

**Affiliations:** Universidad Nacional de Colombia, Fizmako Research Group, Bogotá, Colombia

## Abstract

The Duffing oscillator of a system with strong quadratic damping is considered. We give an elementary approximate analytical solution to this oscillator in terms of exponential and trigonometric functions. We compare the analytical approximant with the Runge–Kutta numerical solution. We also solve the oscillator by menas of He's homotopy method and the famous Krylov–Bogoliubov–Mitropolsky method. The approximant allows estimating the points at which the solution crosses the horizontal axis.

## 1. Introduction

In the standard textbooks, usually the systems with linear damping are considered. Due to their simplicity and the existence of an exact analytical solution, the problem is discussed in details. Unfortunately, in reality, the systems and damping are usually not linear. In recent times, a number of articles have appeared in the literature which deal with the phenomenon of a linear oscillator subject to a quadratic damping force [1–7]. Most elementary textbooks deal with viscous damping for the obvious reason that it involves a linear dependance on the velocity of the oscillator and presents the simplest situation where an exact analytical treatment is possible. In general, this involves the analysis of a second-order ordinary differential equation (ODE) of the Liénard type, namely,

(1) $$\begin{array}{c} \ddot{x}+f\left(x\right)\dot{x}+g\left(x\right)=0. \end{array}$$

Nonlinear equations of motion such as this are seldom addressed in intermediate instruction in classical dynamics; this one is problematic because it cannot be solved in terms of elementary functions. The principal feature associated with quadratic damping is a discontinuous jump of the damping force in the equation of motion whenever the velocity vanishes such that the frictional force always opposes the motion. In this paper, we will consider the following quadratically damped Duffing oscillator ($f\left(x\right)=\varepsilon \left|\dot{x}\right|$ and *g*(*x*)=*αx*+*βx*^3^):

(2) $$\begin{array}{c} \ddot{x}+\varepsilon \dot{x}\left|\dot{x}\right|+\alpha x+\beta x^{3}=0,\;x\left(0\right)=x_{0}\text{ and }x^{\prime }\left(0\right)={\dot{x}}_{0}. \end{array}$$

The quadratically damped oscillator (2) is never critically damped or overdamped, and that to first order in the damping constant, the oscillation frequency is identical to the natural frequency. In the abscence of damping, we obtain the Duffing equation

(3) $$\begin{array}{c} \ddot{x}+\alpha x+\beta x^{3}=0,\;x\left(0\right)=x_{0}\text{ and }x^{\prime }\left(0\right)={\dot{x}}_{0}. \end{array}$$

Equation (3) admits the exact analytical solution [8].

(4) $$\begin{array}{c} x\left(t\right)=\frac{x_{0}cn\left(\sqrt{\omega }t|m\right)+{\overset{˙}{x}}_{0}|\sqrt{\omega }sn\left(\sqrt{\omega }t|m\right)dn\left(\sqrt{\omega }t|m\right)}{1+bsn{\left(\sqrt{\omega }t|m\right)}^{2}}, \end{array}$$

where

(5) $$\begin{array}{c} \omega =\sqrt{\Delta },\\ m=\frac{1}{2}\left(1-\frac{\alpha }{\sqrt{\Delta }}\right),\\ b=\frac{1}{2}\left(\frac{\alpha +\beta x_{0}^{2}}{\sqrt{\Delta }}-1\right),\\ \Delta ={\left(\alpha +\beta x_{0}^{2}\right)}^{2}+2\beta x_{0}^{2}\neq 0. \end{array}$$

## 2. Solution Procedure

In what follows, we will assume that if *x*=*x*(*t*) is a solution to the i.v.p. (2), then

(6) $$\begin{array}{c} \underset{t⟶\infty }{\lim}x\left(t\right)=0. \end{array}$$

Our aim is to give an approximate analytical solution to the i.v.p. (2). The residual function *R*=*R*(*t*) is defined as follows:

(7) $$\begin{array}{c} R\left(t\right)=\ddot{x}+\varepsilon \dot{x}\left|\dot{x}\right|+\alpha x+\beta x^{3}=\ddot{x}+\delta \varepsilon {\dot{x}}^{2}+\alpha x+\beta x^{3},\;\delta =\pm 1. \end{array}$$

### 2.1. First Approach

The ansatz is assumed as

(8) $$\begin{array}{c} x\left(t\right)=c_{0}e^{-\rho t}\cos\left(\sqrt{\omega }t+\;\cos^{-1}\left(\frac{x_{0}}{c_{0}}\right)\right). \end{array}$$

We have

(9) $$\begin{array}{c} R\left(t\right)=\frac{1}{4}c_{0}\cos\left(\theta \right)e^{-\rho t}\left(4\alpha +3\beta c_{0}^{2}e^{-2\rho t}+4\rho^{2}-4\omega \right)+\\ +\frac{1}{4}\beta c_{0}^{3}\cos\left(3\theta \right)e^{-3\rho t}+\frac{1}{2}c_{0}^{2}\delta \varepsilon \;\cos\left(2\theta \right)\left(\rho^{2}-\omega \right)e^{-2\rho t}+\\ +c_{0}^{2}\delta \varepsilon \rho \sqrt{\omega }\sin\left(2\theta \right)e^{-2\rho t}+\frac{1}{2}c_{0}^{2}\delta \varepsilon \left(\rho^{2}+\omega \right)e^{-2\rho t}+2c_{0}\rho \sqrt{\omega }\sin\left(\theta \right)e^{-\rho t},\;\delta =\pm 1. \end{array}$$

Since for small *t*, *e*^−2*ρt*^ ≈ 1, we will choose the value of *ω* so that

(10) $$\begin{array}{c} 4\alpha +3\beta c_{0}^{2}+4\rho^{2}-4\omega =0. \end{array}$$

Then,

(11) $$\begin{array}{c} \omega =\alpha +\frac{3}{4}\beta c_{0}^{2}+\rho^{2}. \end{array}$$

The value of *c*_0_ is found from the initial condition $x^{\prime }\left(0\right)={\dot{x}}_{0}$, and it reads

(12) $$\begin{array}{c} c_{0}=\pm \sqrt{\frac{3\beta x_{0}^{2}-4\alpha -4\rho^{2}\pm \sqrt{16{\left(\alpha +\rho^{2}\right)}^{2}+3\beta \left(8x_{0}^{2}\left(\alpha +3\rho^{2}\right)+3\beta x_{0}^{4}+32\rho {\dot{x}}_{0}x_{0}+16{\dot{x}}_{0}^{2}\right)}}{6\beta }}. \end{array}$$

In the case when *β*⟶0, we define

(13) $$\begin{array}{c} c_{0}=\pm \sqrt{\frac{x_{0}^{2}\left(\alpha +2\rho^{2}\right)+2\rho {\dot{x}}_{0}x_{0}+{\dot{x}}_{0}^{2}}{\alpha +\rho^{2}}}. \end{array}$$

The number *ρ* is a free parameter that is chosen in order to minimize the residual error.

### 2.2. Second Approach

#### 2.2.1. He's Homotopy Method

We will approximate the expression $\varepsilon \dot{x}\left|\dot{x}\right|$ by means of the formula

(14) $$\begin{array}{c} \varepsilon \dot{x}\left|\dot{x}\right|\approx r_{0}\dot{x}+r_{1}{\dot{x}}^{3}+r_{2}{\dot{x}}^{5},\;\left|\dot{x}\right|\leq M, \end{array}$$

where

(15) $$\begin{array}{c} r_{0}=-\frac{\left(\sqrt{3}-3\right)M}{3\sqrt{6}}\varepsilon ,\\ r_{1}=\frac{\sqrt{2/3}\left(8\sqrt{3}-9\right)}{3M}\varepsilon ,\\ r_{2}=-\frac{4\sqrt{2/3}\left(2\sqrt{3}-3\right)}{3M^{3}}\varepsilon . \end{array}$$

The homotopy equation is defined as

(16) $$\begin{array}{c} H\left(x,p\right)=\ddot{x}+\alpha x+p\left[r_{0}\dot{x}+r_{1}{\dot{x}}^{3}+r_{2}{\dot{x}}^{5}+\beta x^{3}\right]. \end{array}$$

Following He's approach [9–16], we assume the solution in the ansatz form

(17) $$\begin{array}{c} x\left(t\right)=\exp\left(-\mu pt\right)\left(r_{0}\cos\left(\omega t+B\right)+py_{1}\left(\omega t+B\right)\right),\;\text{where }\omega =\sqrt{\alpha +p\omega_{1}}. \end{array}$$

Let *τ*=*ωt*+*B*. Then,

(18) $$\begin{array}{c} H\left(x,p\right)=\frac{1}{16}\left(\begin{array}{c} -2\sqrt{\alpha }r_{0}\left(8r_{0}+5\alpha^{2}r_{0}^{4}r_{2}+6\alpha r_{0}^{2}r_{1}-16\mu \right)\sin\left(\tau \right)+\\ 4r_{0}\left(3r_{0}^{2}\beta -4\omega_{1}\right)\cos\left(\tau \right)+\\ \left(5\alpha^{5/2}r_{0}^{5}r_{2}+4\alpha^{3/2}r_{0}^{3}r_{1}\right)\sin\left(3\tau \right)+4r_{0}^{3}\beta \;\cos\left(3\tau \right)-\\ \alpha^{5/2}r_{0}^{5}r_{2}\sin\left(5\tau \right)+16\alpha y_{1}\left(\tau \right)+16\alpha y_{1}^{\prime \prime }\left(\tau \right). \end{array}\right)p+\cdots . \end{array}$$

We define

(19) $$\begin{array}{c} \omega_{1}=\frac{3r_{0}^{2}\beta }{4},\\ \mu =\frac{1}{16}\left(8r_{0}+5\alpha^{2}r_{0}^{4}r_{2}+6\alpha r_{0}^{2}r_{1}\right). \end{array}$$

Then,

(20) $$\begin{array}{c} H\left(x,p\right)=\left(5\alpha^{5/2}r_{0}^{5}r_{2}+4\alpha^{3/2}r_{0}^{3}r_{1}\right)\sin\left(3\tau \right)+4r_{0}^{3}\beta \;\cos\left(3\tau \right)\\ -\alpha^{5/2}r_{0}^{5}r_{2}\sin\left(5\tau \right)+16\alpha y_{1}\left(\tau \right)+16\alpha y_{1}^{\prime \prime }\left(\tau \right). \end{array}$$

Solving the ode *H*(*x*, *p*)=0 for *y*_1_=*y*_1_(*τ*) gives

(21) $$\begin{array}{c} y_{1}\left(\tau \right)=\frac{12r_{0}^{3}\beta \;\cos\left(3\tau \right)+3\alpha^{3/2}r_{0}^{3}\left(5\alpha r_{0}^{2}r_{2}+4r_{1}\right)\sin\left(3\tau \right)-\alpha^{5/2}r_{0}^{5}r_{2}\sin\left(5\tau \right)}{384\alpha }. \end{array}$$

The first-order homotopy approximation will then be

(22) $$\begin{array}{c} \left(r_{0}\cos\left(w\left(t\right)\right)+\frac{1}{384\alpha }\left(\begin{array}{c} 12r_{0}^{3}\beta \;\cos\left(3w\left(t\right)\right)+3\alpha^{3/2}r_{0}^{3}\left(5\alpha r_{0}^{2}r_{2}+4r_{1}\right)\\ \sin\left(3w\left(t\right)\right)-\alpha^{5/2}r_{0}^{5}r_{2}\sin\left(5w\left(t\right)\right) \end{array}\right)\right),\;\text{where }\\ w\left(t\right)=\sqrt{\alpha +\frac{3\beta }{4}r_{0}^{2}}t+B,\;x\left(t\right)=\exp\left(-\frac{1}{16}\left(8r_{0}+6\alpha r_{1}r_{0}^{2}+5\alpha^{2}r_{2}r_{0}^{4}\right)t\right). \end{array}$$

The constants *r*_0_ and *r*_1_ are determined from the initial conditions

(23) $$\begin{array}{c} x\left(0\right)=x_{0},\\ x^{\prime }\left(0\right)={\dot{x}}_{0}. \end{array}$$

### 2.3. Third Approach

#### 2.3.1. The Krylov–Bogoliubov–Mitropolsky Method (KBM)

The Krylov–Bogoliubov–Mitropolsky Method (KBM) is a technique to give an approximate analytical solution to the weakly nonlinear second-order equation

(24) $$\begin{array}{c} \frac{{\text{d}}^{2}u}{\text{d}t^{2}}+\omega_{0}^{2}u=\varepsilon f\left(u,\frac{\text{d}u}{\text{d}t}\right). \end{array}$$

When *ε*=0, the solution of (24) may be expressed as

(25) $$\begin{array}{c} u=a\;\cos\left(\omega_{0}+\theta \right), \end{array}$$

where *a* and *θ* are constants. For the case when *ε* > 0 is small, Krylov and Bogoliubov (1947) assumed that the solution is still given by (25) but with time-varying *a* and *θ* and subject to the condition

(26) $$\begin{array}{c} \frac{\text{d}u}{\text{d}t}=-a\omega_{0}\sin\;\phi ,\;\phi =\omega_{0}t+\theta . \end{array}$$

In the general case, the solution is assumed in the ansatz form

(27) $$\begin{array}{c} u=a\;\cos\;\psi +\sum_{n=1}^{N}\varepsilon^{n}u_{n}\left(a,\psi \right)+O\left(\varepsilon^{N+1}\right), \end{array}$$

where each *u*_*n*_ is a periodic function of *ψ* with a period 2*π* and *a* and *ψ* are assumed to vary with time according to

(28) $$\begin{array}{c} \frac{\text{d}a}{\text{d}t}=\sum_{n=1}^{N}\varepsilon^{n}A_{n}\left(a\right)+O\left(\varepsilon^{N+1}\right),\\ \frac{\text{d}\psi }{\text{d}t}=\omega_{0}+\sum_{n=1}^{N}\varepsilon^{n}\psi_{n}\left(a\right)+O\left(\varepsilon^{N+1}\right). \end{array}$$

In order to uniquely determine *A*_*n*_ and *ψ*_*n*_, we require that no *u*_*n*_ contains cos  *ψ*. Let *N*=3. Then,

(29) $$\begin{array}{c} \frac{\text{d}u}{\text{d}t}=-a\omega_{0}\sin\left(\psi \right)+\left(\omega_{0}u_{1,\psi }-a\psi_{1}\sin\left(\psi \right)+A_{1}\cos\left(\psi \right)\right)\varepsilon +\\ +\left(A_{1}u_{1,a}+\omega_{0}u_{2,\psi }+\psi_{1}u_{1,\psi }-a\psi_{2}\sin\left(\psi \right)+A_{2}\cos\left(\psi \right)\right)\varepsilon^{2}+\\ +\left(A_{2}u_{1,a}+A_{1}u_{2,a}+\omega_{0}u_{3,\psi }+\psi_{2}u_{1,\psi }+\psi_{1}u_{2,\psi }-a\psi_{3}\sin\left(\psi \right)+A_{3}\cos\left(\psi \right)\right)\varepsilon^{3}+\cdots ,\\ \frac{{\text{d}}^{2}u}{\text{d}t^{2}}=-a\omega_{0}^{2}\cos\left(\psi \right)+\left(\omega_{0}^{2}u_{1,\psi \psi }-2a\psi_{1}\omega_{0}\cos\left(\psi \right)-2A_{1}\omega_{0}\sin\left(\psi \right)\right)\varepsilon +\\ +\left(\begin{array}{c} 2A_{1}\omega_{0}u_{1,a\psi }+2\psi_{1}\omega_{0}u_{1,\psi \psi }+\omega_{0}^{2}u_{2,\psi \psi }+\sin\left(\psi \right)\left(-aA_{1}{\dot{\psi }}_{1}-2A_{1}\psi_{1}-2A_{2}\omega_{0}\right)+\\ \cos\left(\psi \right)\left(A_{1}{\dot{A}}_{1}-a\left(2\psi_{2}\omega_{0}+\psi_{1}^{2}\right)\right) \end{array}\right)\varepsilon^{2}+\\ +\left(\begin{array}{c} {\dot{A}}_{1}A_{1}u_{1,a}+A_{1}^{2}u_{1,aa}+2A_{1}\psi_{1}u_{1,a\psi }+2A_{1}\omega_{0}u_{2,a\psi }+2A_{2}\omega_{0}u_{1,a\psi }+\\ A_{1}{\dot{\psi }}_{1}u_{1,\psi }+2\psi_{2}\omega_{0}u_{1,\psi \psi }+2\psi_{1}\omega_{0}u_{2,\psi \psi }+\psi_{1}^{2}u_{1,\psi \psi }+\omega_{0}^{2}u_{3,\psi \psi }+\\ \sin\left(\psi \right)\left(-aA_{2}{\dot{\psi }}_{1}-aA_{1}{\dot{\psi }}_{2}-2A_{2}\psi_{1}-2A_{1}\psi_{2}-2A_{3}\omega_{0}\right)+\\ \cos\left(\psi \right)\left(-2a\psi_{3}\omega_{0}-2a\psi_{1}\psi_{2}+A_{2}{\dot{A}}_{1}+A_{1}{\dot{A}}_{2}\right) \end{array}\right)\varepsilon^{3}+\cdots . \end{array}$$

Here,

(30) $$\begin{array}{c} {\dot{\psi }}_{1}=\psi_{1}^{\prime }\left(t\right),\\ u_{2,\psi }=\frac{\partial u_{2}}{\partial \psi },\\ u_{2,\psi \psi }=\frac{\partial^{2}u_{2}}{\partial \psi^{2}},\\ u_{1,a\psi }=\frac{\partial^{2}u_{1}}{\partial \psi \;\partial a}\text{, etc.} \end{array}$$

Let us consider the i.v.p.

(31) $$\begin{array}{c} \ddot{x}+r_{0}\dot{x}+r_{1}{\dot{x}}^{3}+r_{2}{\dot{x}}^{5}+\alpha x+\beta x^{3}=0,\;x\left(0\right)=x_{0},x^{\prime }\left(0\right)={\dot{x}}_{0}\text{ for }0\leq t\leq T. \end{array}$$

The values for *r*_0_, *r*_1_, and *r*_2_ are given by (15). Using KBM, we obtain the following odes for determining *a*=*a*(*t*) and *ψ*=*ψ*(*t*):

(32) $$\begin{array}{c} \dot{a}=-\frac{1}{2}\varepsilon s_{0}a+\frac{3\varepsilon \left(\beta s_{0}-2\alpha^{2}s_{1}\right)}{16\alpha }a^{3}+\frac{\varepsilon \left(-195\beta^{2}s_{0}-96\alpha^{2}\beta s_{1}-99^{2}\alpha^{3}\varepsilon^{2}s_{0}s_{1}^{2}-320\alpha^{4}s_{2}+160^{2}\alpha^{3}\varepsilon^{2}s_{0}^{2}s_{2}\right)}{1024\alpha^{2}}a^{5}-\\ -\frac{\varepsilon \left(189\beta^{2}s_{1}+117\alpha^{3}\varepsilon^{2}s_{1}^{3}+720\alpha^{2}\beta s_{2}+370\alpha^{3}\varepsilon^{2}s_{0}s_{1}s_{2}\right)}{8192\alpha }a^{7}-\frac{\varepsilon s_{2}\left(1701\beta^{2}+1782\alpha^{3}\varepsilon^{2}s_{1}^{2}+2170\alpha^{3}\varepsilon^{2}s_{0}s_{2}\right)}{73728}a^{9}-\\ -\frac{29875\alpha^{4}\varepsilon^{3}s_{1}s_{2}^{2}}{196608}a^{11}-\frac{34625\alpha^{5}\varepsilon^{3}s_{2}^{3}}{589824}a^{13}. \end{array}$$

(33) $$\begin{array}{c} \dot{\psi }=\frac{8\alpha -\varepsilon^{2}s_{0}^{2}}{8\sqrt{\alpha }}+\frac{3\beta \left(8\alpha -\varepsilon^{2}s_{0}^{2}\right)}{64\alpha^{3/2}}a^{2}+\frac{-15\beta^{2}-57\alpha \beta \varepsilon^{2}s_{0}s_{1}+9\alpha^{3}\varepsilon^{2}s_{1}^{2}+40\alpha^{3}\varepsilon^{2}s_{0}s_{2}}{256\alpha^{3/2}}a^{4}+\\ +\frac{3\left(41\beta^{3}-223\alpha^{3}\beta \varepsilon^{2}s_{1}^{2}-310\alpha^{3}\beta \varepsilon^{2}s_{0}s_{2}+400\alpha^{5}\varepsilon^{2}s_{1}s_{2}\right)}{8192\alpha^{5/2}}a^{6}+\\ +\frac{\alpha^{3/2}\varepsilon^{2}s_{2}\left(-471\beta s_{1}+2200\alpha^{2}s_{2}\right)}{24576}a^{8}+\frac{13091\alpha^{5/2}\beta \varepsilon^{2}s_{2}^{2}}{196608}a^{10}.\\ s_{1}=\frac{\sqrt{2/3}\left(8\sqrt{3}-9\right)}{3M},\\ s_{2}=-\frac{4\sqrt{2/3}\left(2\sqrt{3}-3\right)}{3M^{3}},\;\text{where }M=\underset{0\leq t\leq T}{\max}\left|\dot{x}\left(t\right)\right|. \end{array}$$

(34) $$\begin{array}{c} s_{0}=-\frac{\left(\sqrt{3}-3\right)M}{3\sqrt{6}},\\ s_{1}=\frac{\sqrt{2/3}\left(8\sqrt{3}-9\right)}{3M},\\ s_{2}=-\frac{4\sqrt{2/3}\left(2\sqrt{3}-3\right)}{3M^{3}},\;\text{where }M=\underset{0\leq t\leq T}{\max}\left|\dot{x}\left(t\right)\right|. \end{array}$$

The expression for the KBM solution is

(35) $$\begin{array}{c} x=x\left(t\right)=a\;\cos\left(\psi \right)+\\ +\frac{a^{3}\left(\begin{array}{c} 3072\alpha^{2}\beta -2016a^{2}\alpha \beta^{2}+1251a^{4}\beta^{3}-576\alpha \beta \varepsilon^{2}s_{0}^{2}+2304\alpha^{3}\varepsilon^{2}s_{0}s_{1}-\\ 3456a^{2}\alpha^{2}\beta \varepsilon^{2}s_{0}s_{1}+2592a^{2}\alpha^{4}\varepsilon^{2}s_{1}^{2}-477a^{4}\alpha^{3}\beta \varepsilon^{2}s_{1}^{2}-4756a^{4}\alpha^{3}\beta \varepsilon^{2}s_{0}s_{2}+\\ 4080a^{4}\alpha^{5}\varepsilon^{2}s_{1}s_{2}+321a^{6}\alpha^{4}\beta \varepsilon^{2}s_{1}s_{2}+2000a^{6}\alpha^{6}\varepsilon^{2}s_{2}^{2}+1300a^{8}\alpha^{5}\beta \varepsilon^{2}s_{2}^{2} \end{array}\right)\cos\left(3\psi \right)}{98304\alpha^{3}}+\\ +\frac{a^{3}\varepsilon \left(\begin{array}{c} 884736\alpha \beta s_{0}-1741824a^{2}\beta^{2}s_{0}+1179648\alpha^{3}s_{1}+884736a^{2}\alpha^{2}\beta s_{1}-\\ 943488a^{4}\alpha \beta^{2}s_{1}-221184\alpha^{2}\varepsilon^{2}s_{0}^{2}s_{1}+580608a^{2}\alpha^{3}\varepsilon^{2}s_{0}s_{1}^{2}+\\ 383616a^{4}\alpha^{4}\varepsilon^{2}s_{1}^{3}+1474560a^{2}\alpha^{4}s_{2}+1787904a^{4}\alpha^{3}\beta s_{2}-257184a^{6}\alpha^{2}\beta^{2}s_{2}-\\ 552960a^{2}\alpha^{3}\varepsilon^{2}s_{0}^{2}s_{2}+2572800a^{4}\alpha^{4}\varepsilon^{2}s_{0}s_{1}s_{2}+1939680a^{6}\alpha^{5}\varepsilon^{2}s_{1}^{2}s_{2}+\\ 2790400a^{6}\alpha^{5}\varepsilon^{2}s_{0}s_{2}^{2}+2912400a^{8}\alpha^{6}\varepsilon^{2}s_{1}s_{2}^{2}+1226125a^{10}\alpha^{7}\varepsilon^{2}s_{2}^{3} \end{array}\right)\sin\left(3\psi \right)}{37748736\alpha^{5/2}}-\\ -\frac{a^{7}\left(\begin{array}{c} -144\beta^{3}+3024\alpha^{3}\beta \varepsilon^{2}s_{1}^{2}+2672\alpha^{3}\beta \varepsilon^{2}s_{0}s_{2}-3840\alpha^{5}\varepsilon^{2}s_{1}s_{2}\\ +7548a^{2}\alpha^{4}\beta \varepsilon^{2}s_{1}s_{2}-5200a^{2}\alpha^{6}\varepsilon^{2}s_{2}^{2}+285a^{4}\alpha^{5}\beta \varepsilon^{2}s_{2}^{2} \end{array}\right)\cos\left(7\psi \right)}{4718592\alpha^{3}}-\\ -\frac{a^{7}\varepsilon \left(\begin{array}{c} -1440\beta^{2}s_{1}+1728\alpha^{3}\varepsilon^{2}s_{1}^{3}+3072\alpha^{2}\beta s_{2}-1362a^{2}\alpha \beta^{2}s_{2}+9040\alpha^{3}\varepsilon^{2}s_{0}s_{1}s_{2}\\ +16530a^{2}\alpha^{4}\varepsilon^{2}s_{1}^{2}s_{2}+9200a^{2}\alpha^{4}\varepsilon^{2}s_{0}s_{2}^{2}+30065a^{4}\alpha^{5}\varepsilon^{2}s_{1}s_{2}^{2}+15900a^{6}\alpha^{6}\varepsilon^{2}s_{2}^{3} \end{array}\right)\sin\left(7\psi \right)}{4718592\alpha^{3/2}}+\\ +\frac{\left(a^{9}\alpha \varepsilon^{2}s_{2}\left(2268\beta s_{1}-400\alpha^{2}s_{2}+2477a^{2}\alpha \beta s_{2}\right)\right)\cos\left(9\psi \right)}{7864320}\\ +\frac{a^{9}\varepsilon s_{2}\left(\begin{array}{c} -450\beta^{2}+954\alpha^{3}\varepsilon^{2}s_{1}^{2}+784\alpha^{3}\varepsilon^{2}s_{0}s_{2}+3417a^{2}\alpha^{4}\varepsilon^{2}s_{1}s_{2}\\ +2580a^{4}\alpha^{5}\varepsilon^{2}s_{2}^{2} \end{array}\right)\sin\left(9\psi \right)}{4718592\sqrt{\alpha }}-\\ -\frac{\left(1177a^{11}\alpha^{2}\beta \varepsilon^{2}s_{2}^{2}\right)\cos\left(11\psi \right)}{47185920}-\frac{\left(a^{11}\alpha^{7/2}\varepsilon^{3}s_{2}^{2}\left(3564s_{1}+4915a^{2}\alpha s_{2}\right)\right)\sin\left(11\psi \right)}{113246208}\\ +\frac{\left(1175a^{13}\alpha^{9/2}\varepsilon^{3}s_{2}^{3}\right)\sin\left(13\psi \right)}{792723456}. \end{array}$$

The odes (32) and (33) may be solved numerically. However, since we are interested in obtaining analytical solutions, we may limit ourselves to the following approximate solutions:

(36) $$\begin{array}{c} a=a\left(t\right)=\frac{2A\sqrt{r_{0}}}{\sqrt{4r_{0}e^{r_{0}t}+3\alpha r_{1}A^{2}\left(e^{r_{0}t}-1\right)}},\\ \psi =\psi \left(t\right)=\sqrt{\alpha }t+B-\frac{\beta }{2\alpha^{3/2}r_{1}}\left(r_{0}t+\log\left(e^{r_{0}t}+\frac{3A^{2}\left(e^{r_{0}t}-1\right)\alpha r_{1}}{4r_{0}}\right)\right), \end{array}$$

where the values for *r*_0_, *r*_1_, and *r*_2_ are given by (15). The constants *A* and *B* are determined from the initial conditions.

### 2.4. Fourth Approach

We assume the ansatz

(37) $$\begin{array}{c} x\left(t\right)=c_{0}e^{-\rho t}\cos\left(f\left(t\right)+\;\cos^{-1}\left(\frac{x_{0}}{c_{0}}\right)\right),\;f\left(0\right)=0. \end{array}$$

We have

(38) $$\begin{array}{c} R\left(t\right)=\frac{1}{4}c_{0}\exp\left(-3\rho t\right)\\ \left[\begin{array}{c} \left(4e^{2\rho t}\alpha +3c_{0}^{2}\beta +4e^{2\rho t}\rho^{2}-4e^{2\rho t}f^{\prime }{\left(t\right)}^{2}\right)\cos\left(\theta \right)+\\ 2c_{0}e^{\rho t}\delta \varepsilon \rho^{2}+c_{0}^{2}\beta \;\cos\left(3\theta \right)+2c_{0}e^{\rho t}\delta \varepsilon f^{\prime }{\left(t\right)}^{2}+2c_{0}e^{\rho t}\delta \varepsilon \;\cos\left(2\theta \right)\left(\rho^{2}-f^{\prime }{\left(t\right)}^{2}\right)+\\ 4e^{2\rho t}\left(2\rho f^{\prime }\left(t\right)-f^{\prime \prime }\left(t\right)\right)\sin\left(\theta \right)+4c_{0}e^{\rho t}\delta \varepsilon \rho f^{\prime }\left(t\right)\sin\left(2\theta \right) \end{array}\right],\\ \delta =\pm 1. \end{array}$$

We will choose the function *f*=*f*(*t*) so that

(39) $$\begin{array}{c} 4e^{2\rho t}\alpha +3c_{0}^{2}\beta +4e^{2\rho t}\rho^{2}-4e^{2\rho t}f^{\prime }{\left(t\right)}^{2}=0,\;f^{\prime }\left(t\right)>0. \end{array}$$

Then,

(40) $$\begin{array}{c} f\left(t\right)=F\left(t\right)-F\left(0\right),\\ F\left(t\right)=-\frac{\sqrt{4\left(\alpha +\rho^{2}\right)+3\beta c_{0}^{2}e^{-2\rho t}}}{2\sqrt{\beta }c_{0}\rho \sqrt{\left(4\left(\alpha +\rho^{2}\right)e^{2\rho t}/\beta c_{0}^{2}\right)+3}}\\ \left(\sqrt{\beta }c_{0}\sqrt{\frac{4\left(\alpha +\rho^{2}\right)e^{2\rho t}}{\beta c_{0}^{2}}+3}-2\sqrt{\alpha +\rho^{2}}e^{\rho t}\sinh^{-1}\left(\frac{2\sqrt{\alpha +\rho^{2}}e^{\rho t}}{\sqrt{3}\sqrt{\beta }c_{0}}\right)\right). \end{array}$$

The value of *c*_0_ is found from the initial condition $x^{\prime }\left(0\right)={\dot{x}}_{0}$, and it reads

(41) $$\begin{array}{c} c_{0}=\pm \sqrt{\frac{3\beta x_{0}^{2}-4\alpha -4\rho^{2}\pm \sqrt{16\rho^{4}+8\rho^{2}\left(4\alpha +9\beta x_{0}^{2}\right)+{\left(4\alpha +3\beta x_{0}^{2}\right)}^{2}+96\beta \rho x_{0}{\dot{x}}_{0}+48\beta {\dot{x}}_{0}^{2}}}{6\beta }}. \end{array}$$

In the case when *β*⟶0, we define

(42) $$\begin{array}{c} c_{0}=\pm \sqrt{\frac{1}{2}\left(x_{0}^{2}+\frac{x_{0}^{2}\left(\alpha +3\rho^{2}\right)+4\rho {\dot{x}}_{0}x_{0}+2{\dot{x}}_{0}^{2}}{\left|\alpha +\rho^{2}\right|}\right)}. \end{array}$$

## 3. Analysis and Discussion

In this section, we will compare the accuracy of the solution methods using the different approaches described in the previous section.

**Example 1 .**

Let us consider the i.v.p.

(43) $$\begin{array}{c} \ddot{x}+0.2\dot{x}\left|\dot{x}\right|+x+2x^{3}=0,\;x\left(0\right)=0,\;x^{\prime }\left(0\right)=0.1. \end{array}$$

The approximate analytical solution using the first approach (see formula (8)) for *ρ*=0.007191 is

(44) $$\begin{array}{c} x_{\text{approx}}\left(t\right)=0.0992665e^{-0.007191t}\sin\left(1.00739t\right). \end{array}$$

**Figure 1:**
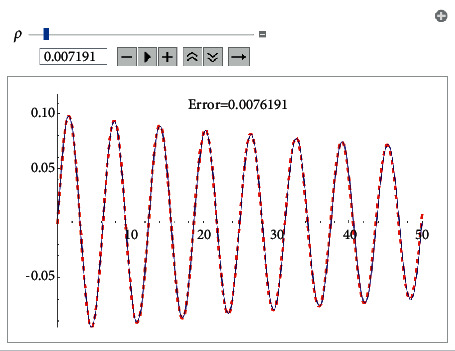
Comparison between the analytical approximant (44) (formula (8)) and the Runge–Kutta numerical solution for the i.v.p (43).

It is shown in Figure 1.

**Figure 2:**
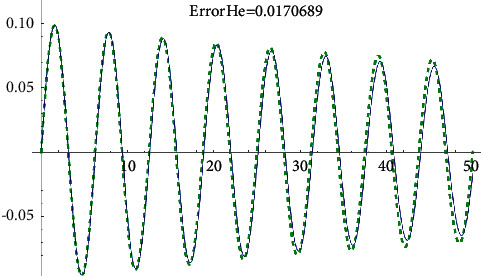
He's homotopy method-error comparison with the Runge–Kutta numerical solution.

The solution obtained by means of He's homotopy method (see Figure 2) equals

(45) $$\begin{array}{c} x_{\text{He}}\left(t\right)=e^{-0.0089515t}\left(\begin{array}{c} -0.0000427888\;\sin\left(3.00025\left(t+7.85294\right)\right)-3.49e-\\ 6\;\sin\left(5.00041\left(t+7.85294\right)\right)-0.0999944\;\cos\left(1.00008\left(t+7.85294\right)\right)\\ -1.03e-6\;\cos\left(3.00025\left(t+7.85294\right)\right)-6.9e-8\;\cos\left(5.00041\left(t+7.85294\right)\right) \end{array}\right). \end{array}$$

**Figure 3:**
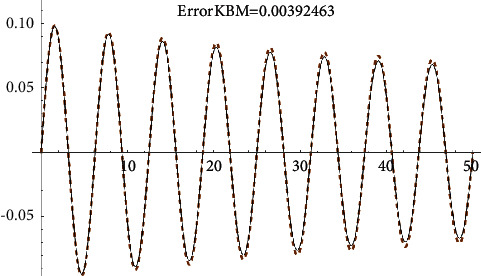
KBM-error comparison with the Runge–Kutta numerical solution.

Using the KBM, we obtained the following solution (Figure 3):

(46) $$\begin{array}{c} x_{\text{KBM}}\left(t\right)=\frac{\begin{array}{c} 6.736e-9\;\sin\left(\begin{array}{c} -1.89144\;\log\left(0.850343-1.e^{0.00345092t}\right)+\\ \left(4.2634+5.94212i\right)-4.99347t \end{array}\right)+\left(1.32e-6-1.43e-6e^{0.00345092t}\right)\\ -\sin\left(-1.13486\;\log\left(0.850343-1.e^{0.00345092t}\right)+\left(2.55804+3.56527i\right)-2.99608t\right)\\ \left(1.1e-6e^{0.00345092t}-9.2e-7\right)\cos\left(\begin{array}{c} -1.13486\;\log\left(0.850343-1.e^{0.00345092t}\right)+\\ \left(2.55804+3.56527i\right)-2.99608t \end{array}\right)+\\ \left(0.00844854-0.0198709e^{0.00345092t}+0.0116841e^{0.00690184t}\right),\\ \cos\left(-0.378287\;\log\left(0.850343-1.e^{0.00345092t}\right)+\left(0.85268+1.18842i\right)-0.998695t\right) \end{array}}{\sqrt{0.0922354e^{0.00345092t}-0.0784317}{\left(0.850343-e^{0.00345092t}\right)}^{2}}. \end{array}$$

**Figure 4:**
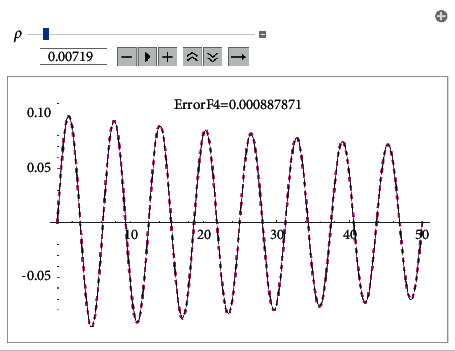
Method using the fourth approach-error comparison with the Runge–Kutta numerical solution.

Now, using the fourth approach, we get the solution (see Figure 4)

(47) $$\begin{array}{c} x_{\text{trig}}\left(t\right)=-0.0992665e^{-0.00719t}\\ \sin\left(\frac{\begin{array}{c} 69.541\sqrt{4.00021+0.0591231e^{-0.01438t}}-\\ 4.8811e^{0.00719t}\sqrt{202.977e^{0.01438t}+3}\sqrt{4.00021+0.0591231e^{-0.01438t}}\sinh^{-1}\left(8.22551e^{0.00719t}\right) \end{array}}{0.01478+1.e^{0.01438t}}+249.895\right). \end{array}$$

**Example 2 .**

Let us consider the i.v.p.

(48) $$\begin{array}{c} \ddot{x}+0.2\dot{x}\left|\dot{x}\right|+x+10x^{3}=0,\;x\left(0\right)=0,\;x^{\prime }\left(0\right)=0.1. \end{array}$$

The approximate analytical solution for *ρ*=0.0084 using the fourth approach is

(49) $$\begin{array}{c} x_{\text{approx}}\left(t\right)=\\ 0.097e^{-0.0084t}\sin\left(\begin{array}{c} 59.5231\\ \left(\begin{array}{c} 2.067-\sqrt{4.0003+0.280334e^{-0.017t}}-\\ 0.611395\left(\begin{array}{c} 6.672-\\ \frac{e^{0.0084t}\sqrt{42.81e^{0.017t}+3}\sqrt{4.0003+0.28e^{-0.017t}}\sinh^{-1}\left(3.778e^{0.0084t}\right)}{0.28+4.0003e^{0.017t}} \end{array}\right) \end{array}\right) \end{array}\right). \end{array}$$

**Figure 5:**
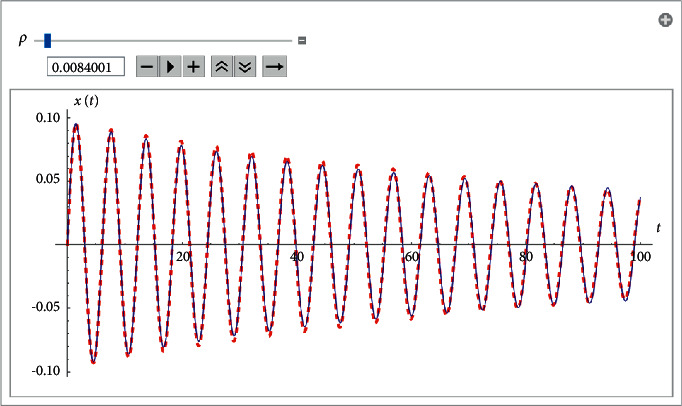
Comparison between the analytical approximant (49) and the Runge–Kutta numerical solution for the i.v.p (48).

It is shown in Figure 5.

The obtained results may be applied to solve the pendulum equation with quadratic damping

(50) $$\begin{array}{c} \ddot{x}+\varepsilon \dot{x}\left|\dot{x}\right|+\omega^{2}\sin\;x=0,\;x\left(0\right)=x_{0},\;x^{\prime }\left(0\right)={\dot{x}}_{0}. \end{array}$$

Indeed, we may use the approximation

(51) $$\begin{array}{c} \sin\;x\approx x-\frac{3}{19}x^{3}\text{ for }\left|x\right|\leq \frac{\pi }{3}, \end{array}$$

and then, we replace i.v.p. (43) with the i.v.p.

(52) $$\begin{array}{c} \ddot{x}+\varepsilon \dot{x}\left|\dot{x}\right|+\omega^{2}x-\frac{3\omega^{2}}{19}x^{3}=0,\;x\left(0\right)=x_{0},\;x^{\prime }\left(0\right)={\dot{x}}_{0}. \end{array}$$

## 4. Conclusions

We have obtained approximate analytical solutions to the quadratically damped Duffing oscillator equation by means of an elementary approach. We introduced a *ρ*−parameter technique that allowed us to optimize the obtained solution. The results are also valid for the linear quadratically damped oscillator $\ddot{x}+\varepsilon \dot{x}\left|\dot{x}\right|+\alpha x=0$. A similar approach may be employed to study the quadratically damped cubic-quintic oscillator $\ddot{x}+\varepsilon \dot{x}\left|\dot{x}\right|+\alpha x+\beta x^{3}+\gamma x^{5}=0$. Also, a more general quadratically damped oscillator $\ddot{x}+\varepsilon \dot{x}\left|\dot{x}\right|+h\left(x\right)=0$ may be solved for any odd parity function *h*(*x*). In future work, we will study quadratically damped forced oscillators having the form $\ddot{x}+\varepsilon \dot{x}\left|\dot{x}\right|+h\left(x\right)=F\left(t\right)$ for any continuous functions *h*(*x*) and *F*(*t*).

## Data Availability

No data were used to support this study.
